# *Paratylenchus Ilicis* n. Sp. (Nematoda: Paratylenchinae) Associated with Holly from the Netherlands and New Taxonomical and Phylogenetic Support for the Synonymization of *Cacopaurus* with *Paratylenchus*

**DOI:** 10.2478/jofnem-2022-0037

**Published:** 2022-09-24

**Authors:** Phougeishangbam Rolish Singh, Bram Lokker, Marjolein Couvreur, Wim Bert, Gerrit Karssen

**Affiliations:** 1 Department of Biology, Nematology Research Unit, Ghent University, K. L. Ledeganckstraat 35, 9000 Ghent, Belgium; 2 National Plant Protection Organization, Geertjesweg 15, 6706EA Wageningen, The Netherlands

**Keywords:** 18S, *Cacopaurus*, *cox1*, D2–D3, Hilversum, holly, *Ilex aquifolium*, ITS, morphology, *Paratylenchus ilicis*, phylogeny, scanning electron microscopy, systematics, taxonomy

## Abstract

*Paratylenchus ilicis* n. sp. was found associated with holly in the Netherlands and was described based on morphology, morphometrics, rRNA and mitochondrial *cox1* genes, phylogenetic relationships with other *Paratylenchus* species, host information and geographical distribution. This species can be morphologically diagnosed based on its light brown, slightly obese to obese females with tubercles on cuticle, lateral bands widening into an ovoid field around vulva level, stylet length of 70 μm to 100 μm, outstretched to reflexed ovary, rounded sperm-filled spermatheca, vagina opening into a thick-walled rounded space, absence of vulval flaps and vulva at 89% to 95% of body length, very short tail in all life stages, and a characteristic finger-like tail tip in juveniles (J2). The new species is morphologically closest to *Cacopaurus pestis* but differs based on the absence of a scutellum-like differentiation in the lateral field, the ovaries that can be outstretched or reflexed, and the finger-like tail tip in J2. Furthermore, both species were found to be molecularly distant from one another, found in different habitats, and are associated with different hosts. The high morphological similarity between *Cacopaurus* and *Paratylenchus* and our phylogenetic analyses, revealing that the former is embedded within different *Paratylenchus* clades and thus polyphyletic, provide new evidences for the synonymization of *Cacopaurus* with *Paratylenchus*.

In the plant-parasitic nematode (PPN) family Tylenchulidae Skarbilovich, 1947, *Paratylenchus* Micoletzky, 1922 is a large genus with 140 nominal species described to date that are morphologically diverse, and distributed worldwide (Ghaderi *et al*., 2016; Clavero-Camacho *et al*., 2021a, 2021b; Singh *et al*., 2021; Palomares-Rius *et al*., 2022). Within this nematode family, *Cacopaurus* Thorne, 1943 has been described as a monotypic genus with *C*. *pestis* Thorne, 1943 as the only species, which is an important parasite of *Juglans regia* L. (Persian walnut). *Cacopaurus* has been separated from its closest genus *Paratylenchus*, based on the obese and distorted body of its female, the tubercles formed on the annules of female cuticle, and the sessile type of plant parasitism (Thorne, 1943). However, Goodey (1963) synonymized *Cacopaurus* with *Paratylenchus* because of the absence of consistent differential traits, apart from the fact that females of *Cacopaurus* are sessile and swollen. Nevertheless, *C*. *pestis* continued to receive recognition in subsequent works of many nematode taxonomists including Raski (1991), Raski and Luc (1987), Ebsary (1991), Siddiqi (2000), Andrássy (2007), and Ghaderi *et al*. (2014, 2016). The first molecular data of *C*. *pestis* were recently generated from seven Iranian populations included in the study of Mokaram Hesar *et al*. (2019), wherein 14 sequences of 28S rRNA (MK506797–MK506803) and ITS rRNA (MK506784–MK506790) genes from all the seven populations were published. Interestingly, based on the phylogenetic trees of Tylenchulidae reconstructed using both rDNA sequences in the above-mentioned study, the *C*. *pestis* sequences were found embedded within clades of *Paratylenchus*, reinforcing the argument of Goodey against the separation of the two (Goodey, 1963; Mokaram Hesar *et al*., 2019).

*Cacopaurus pestis* has been considered an important plant parasite responsible for the slow decline disease of approximately 35-yr-old walnut trees in a California orchard, where the onset of symptoms of declining trees, fruit reduction, and eventual dying of the trees took several years (Thorne, 1943). This species was further reported in France (Scotto La Massese, 1971), Italy (Inserra, 1973), Spain (Bello and Belart, 1975), and Iran (Sturhan, 1977; Gharahkhani *et al*., 2007; Bahmani *et al*., 2013; Mokaram Hesar *et al*., 2019), being found associated with different plants such as *Citrus aurantium* L. (sour orange), *Crataegus* sp. (hawthorn), *Populus nigra* L. (poplar), *Rosa indica* L. cv Major (rose), *Syringa vulgaris* L. (lilac), and *Ulmus* sp. (elm). However, *C*. *pestis* has never been reported so far from the northern parts of Europe.

In the current work, a *Paratylenchus* population was uncovered from Hilversum of the Netherlands from rhizosphere and roots of a holly plant (*Ilex aquifolium* L.). This population closely resembled *C*. *pestis* in its morphology; however, its molecular information based on rRNA and *cox1* sequences appeared new. Hence, this paper aimed at describing the uncovered species as *Paratylenchus ilicis* n. sp. based on morphology (light microscopy, scanning electron microscopy (SEM), illustrations, and morphometrics), molecular data (D2–D3 of 28S, 18S, ITS rRNA genes and *cox1* of mtDNA) and subsequent phylogenetic analysis, host information, and geographical locations, and also to support the synonymization of *Cacopaurus* with *Paratylenchus* using additional arguments based on our integrated data analyses.

## Materials and Methods

### Nematode extraction

During a survey of root-knot nematodes, several soil samples were collected from rhizospheres of holly (*I. aquifolium* L.) from Hilversum, the Netherlands, including root materials of the hosts. Nematodes were extracted from the soil and the roots following centrifugal flotation method (van Bezooijen, 2006) at the National Plant Protection Organization (NPPO), Wageningen, the Netherlands. The nematode extract was stored at 4°C during the course of the study.

### Morphological characterization

Specimens of *Paratylenchus* collected from the extracts were picked out separately in a drop of water in glass cavity blocks and killed and fixed using hot 4% formaldehyde (about 60°C). The specimens were left in the fixative for 7 d at 4°C, then gradually transferred to anhydrous glycerin following the protocol described in Seinhorst (1959), and subsequently mounted on glass slides in glycerin medium. Morphological characterization of the nematodes was done using an Olympus BX51 DIC Microscope (Olympus Optical, Tokyo, Japan), equipped with an Olympus C5060Wz camera and a drawing tube (Singh *et al*., 2018). Illustrations were improved using (Adobe Photoshop CS6, San Jose, CA).

For SEM, fixed specimens were washed in 0.1 M phosphate buffer (pH = 7.5) and dehydrated in a graded series of ethanol solutions, critical-point-dried with liquid CO_2_, mounted on stubs with carbon tabs (double conductive tapes), coated with gold of 25 nm, and photographed with a JSM-840 EM (JEOL, Tokyo, Japan) at 12 kV (Singh *et al*., 2018).

### Molecular characterization

Prior to DNA extraction, individual nematodes were temporarily mounted on glass slides in tap water and heat relaxed until they stopped movement. Morphological vouchers of the specimens were made using the above-mentioned camera equipped microscope, and then recovered in distilled water for further molecular characterization (Singh *et al*., 2021). The cuticle of each nematode was punctured using a metallic pin (used as worm-picking tool) and individually transferred to a PCR tube containing 20 ml of worm lysis buffer (50 mM KCl, 10 mM Tris at pH = 8.3, 2.5 mM MgCl_2_, 0.45% NP 40 [Tergitol Sigma Belgium], and 0.45% Tween 20). The PCR tubes were then frozen at –20°C (10 min) followed by adding 1 ml proteinase K (1.2 mg/ml), incubation at 65°C (1 h) and 95°C (10 min), and finally centrifuging the lysate at 14,000 rcf for 1 min (Singh *et al*., 2018).

PCR amplifications of ITS and D2-D3 of 28S rRNA genes were done using the primer pairs, Vrain2F: 5´-CTT TGT ACA CAC CGC CCG TCG CT-3´/Vrain2R: 5´-TTT CACT CGC CGT TAC TAA GGG AAT C-3´ (Vrain *et al*., 1992) and D2A: 5´-ACA AGT ACC GTG AGG GAA AGT TG-3´/D3B: 5´-TCC TCG GAA GGA ACC AGC TAC TA-3´ (Nunn, 1992), respectively, following the thermal profiles described in Singh *et al*. (2019). For amplification of partial sequence of the mitochondrial *cox1* gene, the primer pair JB3: 5´-TTT TTT GGG CAT CCT GAG GTT TAT-3´/JB4.5: 5´-TAA AGA AAG AAC ATA ATG AAA ATG-3´ was used according to Bowles *et al*. (1992). The PCR products were enzymatically cleaned with alkaline phosphatase (1 U/ml) and exonuclease I (20 U/ml) for 15 min at 37°C followed by 15 min at 80°C (Singh *et al*., 2020), and then sent for sequencing at Macrogen (https://dna.macrogen.com) The contigs were made from the newly produced forward and backward sequences using Geneious Prime 2020.0.5 (https://www.geneious.com) and were deposited in GenBank.

### Phylogenetic analyses

The obtained rRNA and *cox1* gene sequences were analyzed with other relevant sequences available in GenBank using programs in Geneious Prime 2020.0.5. Multiple alignments of DNA sequences were made using MUSCLE in Geneious Prime 2020.0.5 with the default parameters and followed by manually trimming off the poorly aligned ends. Bayesian phylogenetic analysis (using MrBayes 3.2.6) was carried out using the GTR + I + G nucleotide substitution model; analyses were run under 1 × 10^6^ generations (four runs), Markov chains were sampled every 100 generations, and 20% of the converged runs were regarded as burnin (Huelsenbeck and Ronquist, 2001). Available sequences of nematode species from different genera, but within the same family (for D2–D3 and ITS) or superfamily (for *cox1*), were selected as outgroups.

## Results and Description

### Systematics

**Figure 1:**
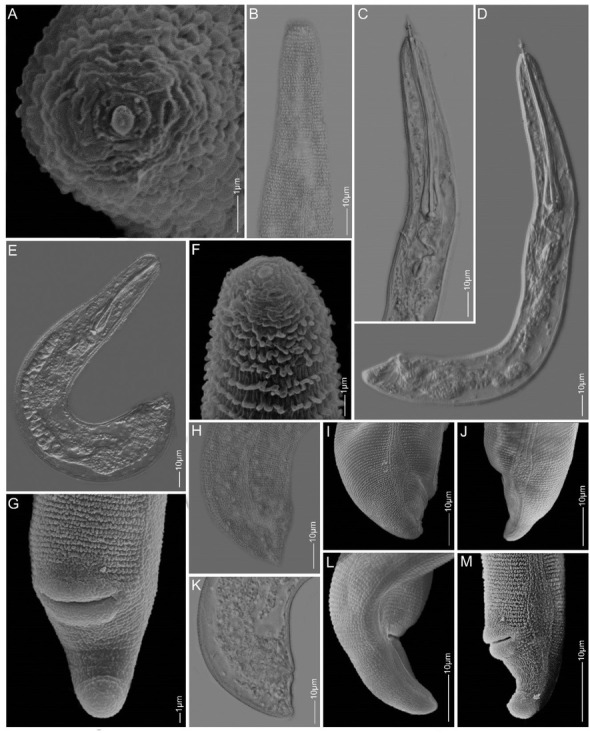
Light and SEM images of *Paratylenchus ilicis* n. sp. paratype females. A: *En face*; B, C, F: Anterior regions showing cuticular ornamentation, stylet, pharynx, and SE pore position; D–E: Total body of (slightly) obese bodies showing major internal structures; G–M: Tail regions showing lateral field, vulva, and tail termini. SE pore: secretory-excretory pore; SEM: scanning electron microscopy.

**Figure 2:**
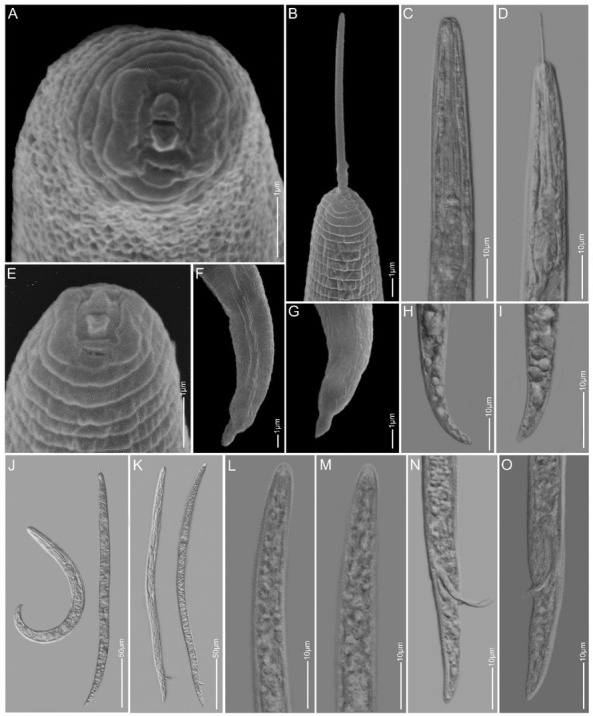
Light and SEM images of *Paratylenchus ilicis* n. sp. paratype juveniles (J2) and males. A, E: *en face* of J2; B–D: Anterior regions showing stylet and pharynx of J2; F–I: Tail regions showing characteristic finger-like tip of J2; J: Total bodies of J2; K: Total bodies of males; L–M: Anterior regions of males showing absence of stylet; N–O: Tail regions showing spicules. SEM: scanning electron microscopy.

**Figure 3:**
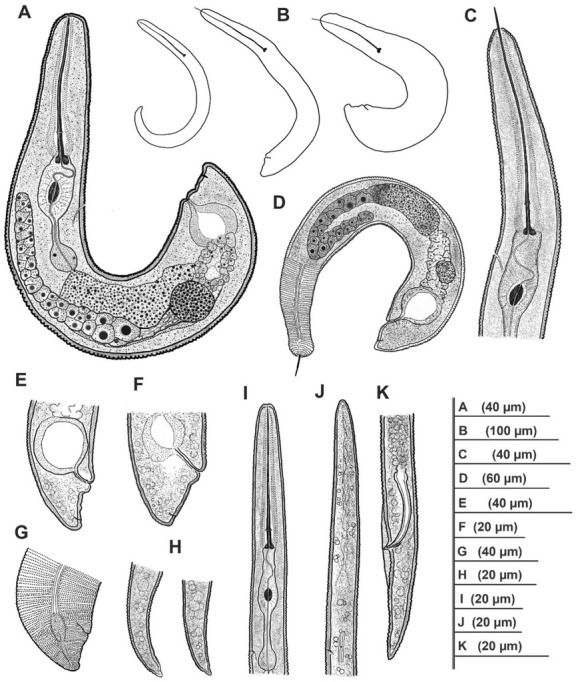
Line illustrations of *Paratylenchus ilicis* n. sp. paratypes. A, B, D: Total bodies showing developmental stages from juvenile (J2) to slightly obese to fully obese females; C: Anterior region of female showing stylet, pharynx, and SE pore position; E–G: Tail regions showing vulva, lateral field differentiation, tail shape, and tips of females; H: Tails of J2; I: Anterior region of J2; J: Anterior region of male; K: Posterior region of male. SE pore: secretory-excretory pore.

*Paratylenchus ilicis* n. sp. Figures 1–3, Table 1

**Table 1:** Morphometrics of *Paratylenchus ilicis* n. sp. females from fixed specimens and mounted in glycerin medium.

Characters	Females	
	Slightly obese	Obese
n	5	10
L	187 ± 23.6 (165–219)	239 ± 22.6 (213–288)
a	10.1 ± 1.0 (9.0–11.1)	7.6 ± 0.7 (6.7–8.8)
b	1.7 ± 0.5 (1.3–2.2)	–
Stylet length	89.3 ± 7.6 (84.1–98.0)	81.4 ± 8.0 (70.2–92.0)
Conus length	77.7 ± 8.8 (72.4–87.8)	65.5 ± 7.5 (60.4–74.1)
Conus % of stylet	86.9 ± 2.6 (84.5–89.6)	84.2 ± 4.5 (79.2–87.5)
Knobs height	3.7 ± 0.6 (3.0–4.2)	3.3 ± 1.2 (2.0–4.2)
Knobs width	5.5 ± 0.4 (5.0–6.0)	5.2 ± 0.7 (4.4–5.8)
Anterior to mid-valve length	86.4 ± 19.4 (68.4–107)	66.7 ± 13.3 (54.2–84.7)
Pharynx length	116 ± 19.8 (99.4–138)	–
Anterior to vulva length	170 ± 20.6 (151–198)	220 ± 21.8 (200–268)
V%	91.1 ± 0.5 (90.4–91.6)	92.2 ± 1.7 (89.2–94.6)
Body width at vulva	16.5 ± 1.8 (14.8–18.7)	22.3 ± 3.2 (18.7–28.7)
Maximum body width	18.4 ± 1.0 (17.3–19.8)	31.5 ± 1.8 (29.7–34.9)

All measurements are in micrometers and presented in the form of average ± SD. (min–max).

### Description

#### Female

Body slender to obese, open C- to J-shaped or folded, obese light brown in color. Lateral field with four distinct lines, each line formed by rows of minute elements, extending from around mid-neck region to terminus, forming three bands; mid-band slightly wider than outer two bands. Lateral field enlarged into a characteristic oval to spindle-shaped structure around vulva level and demarcation of bands lost in this structure. Cuticle 2 mm to 3 mm thick; annuli ornamented by rows of minute refractive tubercles throughout body. Deirids visible in some specimens just around start of lateral field differentiation. Lip region conicaltruncate to rarely rounded and continuous. *En face* roughly square-shaped with weak, non-protruding submedian lobes, and slit-like amphidial fovea on lateral sides of oral opening. Stylet thin and slender, variable in length from 70 mm to almost 100 mm, conus 80% to 90% of stylet length, with slightly posteriorly sloping transversely ovoid knobs. Pharynx often more than half of total body length in slightly obese individuals; median bulb pyriform with large sclerotized valves; isthmus slender and basal bulb rounded to oval, always smaller than median bulb. Secretory-excretory pore (SE pore) variable in position, depending on level of maturity and whether or not stylet is protruded (usually at half way from knobs to valve of median bulb in slightly obese individuals with non-protruded stylet); however, in markedly obese females, SE pore was difficult to observe. Hemizonid not observed. Reproductive system monodelphic with outstretched to sometimes reflexed ovary containing developing oocytes. Spermatheca rounded and filled with sperm cells. Vagina oblique and enlarges into a spacious thick-walled rounded uterine chamber. Vulval lips swelling both anteriorly and posteriorly, no vulva flaps, and vulva located at 89% to 95% of body length from anterior end. Post-uterine sac absent. Tail conical, often with an impression of immediate depression after swelling of posterior vulval lip. Anus obscure, very close to tail tip, 3 mm to 5 mm from tip. Tail terminus bluntly rounded.

#### Male

Body slender and slightly ventrally arcuate when heat relaxed. Cuticular ornamentation not seen. Lip region conical. Anterior part of body poorly developed

without apparent stylet and pharynx. SE pore just above hemizonid. Deirids regularly observed near hemizonid level. Spicules ventrally arcuate, about 15 mm; gubernaculum slightly arcuate, about 4 mm in length. Narrow and thin bursa present, but can be easily overlooked. Cloacal sheath protruding. Tail region narrow, conical, and with finely rounded terminus.

#### Juvenile

Body slender, slightly ventrally arcuate to open C-shaped when heat relaxed. Cuticular ornamentation not seen. Deirids observed near hemizonid level and SE pore just above, or at same level as hemizonid in J2. Lateral field with four lines. Lip region conicaltruncate, submedian lobes not protruding in lateral view. *En face* square-shaped with two thick lateral ridges around slit-like stoma and amphidial fovea next to the ridges, and the four non-protruding submedian lobes in the corners. Stylet well developed, thin and slender, 38 mm to 43 mm in length in younger juveniles (most probably J2) and up to almost 60 mm in older, slightly bigger juveniles (most probably J4). Tail very short (6 mm to 7 mm in J2), conical, and with a characteristic triangular-shaped, finger-like or nipple-like hyaline tip of 3 mm to 4 mm in length.

### Diagnosis and relationships

*Paratylenchus ilicis* n. sp. is characterized by slightly to fully obese females of light brown color with small body of 0.16 mm to 0.29 mm in length and 17 mm

to 35 mm in width. Bodies are ‘J’ or ‘C’ shaped or distorted or folded upon heat relaxation. The cuticle is up to 3 mm thick and refractive tubercles are present on cuticle throughout the body. The lateral field has three bands formed by four lines, and these bands widen into a characteristic ovoid to spindle-shaped field around vulva level. Deirids may be seen at the start of lateral field differentiation. The lip region is conical-truncate to occasionally rounded and is not offset, and the submedian lobes are not seen protruding. The stylet length is variable in length, between 70 mm and 100 mm long, its conus 80% to 90% of the total length. The reproductive tract is monodelphic with an outstretched to a reflexed ovary, a rounded sperm-filled spermatheca, and an oblique vagina which opens into a thick-walled, rounded uterine space. The relative position of vulva is at 89% to 95% of body length from the anterior end and both vulva lips are swollen and without vulva flaps. The tail is very short with a bluntly rounded terminus. The juvenile stylet length is variable (38 mm to 60 mm) depending on the life stage. The younger juvenile (J2) tail terminus has a characteristic finger-like tip. The males do not have a stylet. Spicules and gubernaculum are, respectively, about 15 mm and 4 mm long, and with a very thin bursa, which may be missed during observation. In both the juveniles and males, the SE pore is seen just above the hemizonid, and distinct and rather large deirids are present around the level of hemizonid.

*Paratylenchus ilicis* n. sp. is morphologically very similar to *C*. *pestis*. Both species have females that are slightly to fully obese, similar lateral field differentiation, cuticular ornamentation throughout the body, variable stylet length, a monodelphic reproductive track, sperm-filled spermatheca, spacious thick-walled rounded uterine chamber, swollen vulval lips, absence of vulva flaps, similar relative position of vulva, and similar tail shape and tip. However, the maximum body width of *P. ilicis* n. sp. females were found to be smaller compared to all the records for *C*. *pestis*, including type material (30 mm to 35 mm vs. 35 mm to 43 mm, compiled in Ghaderi *et al*., 2016). In the original description of *C*. *pestis*, a circular organ reminiscent of the scutellum of *Hoplolaimus* von Daday, 1905 was well illustrated in the oval-shaped lateral field differentiation. However, such structure is not observed in females of *P. ilicis* n. sp. The new species has both outstretched and reflexed ovaries, while *C*. *pestis* was always reported with flexures in the female ovaries. The juveniles of *P. ilicis* n. sp. have the characteristic finger-like tail tip while the juveniles of *C*. *pestis* have a bluntly rounded tail tip.

Females of the new species can also be easily separated from that of the molecularly closely related *P. idalimus* (Raski, 1962) Siddiqi and Goodey, 1964 and *P. verus* (Brzeski, 1995) Brzeski, 1998 (see molecular characterization part below) based on body (slender to obese vs. always slender), cuticle (ornamented with tubercles vs. non-ornamented), vulva flaps (absent vs. present but strongly reduced), rounded uterine chamber (present vs. absent), modification of lateral field at vulva level (into a spindle-shaped structure vs. no modification), V% (89% to 95% vs. 71% to 79%), and tail (very short vs. comparatively longer).

*C. pestis* was described from an orchard near Santa Clara, California, associated with Persian walnut (*J. regia* L.). It was subsequently reported in the southern parts of Europe and in Iran, found associated with the same and also newer hosts (Scotto La Massesee, 1971; Inserra, 1973; Bello and Belart, 1975; Sturhan, 1977; Inserra and Vovlas, 1981; Gharahkhani *et al*., 2007; Bahmani *et al*., 2013; Mokaram Hesar *et al*., 2019). However, this species has never been reported from the northern temperate regions. On the other hand, *P. ilicis* n. sp. was found in northern Europe (Hilversum, the Netherlands) with an annual temperature range of 0°C to 22°C and associated with the host plant holly (*I. aquifolium* L.). Most importantly, the rDNA and mtDNA fragments for the new species were found to be very different (see in molecular characterization part below) from that of the Iranian populations of *C*. *pestis* (Mokaram Hesar *et al*., 2019) but found close to those of *P*. *idalimus* and *P*. *verus*, other species with exceptionally long stylets, but without cuticular ornamentations (Ghaderi *et al*., 2016; Singh *et al*., 2021).

## Etymology

The name of the new species is derived from the host plant *I. aquifolium* L., ilicis being the conjugation of ilex.

## Type host and locality

*Paratylenchus ilicis* n. sp. was found in the rhizosphere and on the roots of an *I. aquifolium* L. plant at Hilversum, the Netherlands (GPS coordinates: latitude 52.234481, longitude 5.175350).

## Type material

Female holotype slide (WT3838) and paratype slides of all life stages (WT3839–WT3846) were deposited at Nematode Collection of the National Plant Protection Organization, Wageningen, the Netherlands. One slide (UGMD_104436) containing two females, three juveniles, and one male paratype was deposited at Ghent University Museum, Zoology Collections, Belgium. One slide containing seven paratypes was deposited at UGent Nematode Collection (Slide: UGnem-306) of Nematology Research Unit of Ghent University, Belgium. One slide (FNCT3723) containing two paratype females, three juveniles, and one male was also deposited at the British Plant Nematode Collection at Fera, York, United Kingdom. The LSID code of the new species is urn:lsid:zoobank.org:pub:A49200FF-14AA-4165-B700-D20A1823B208

## Molecular characterization

Four D2–D3 regions of 28S (ON668067–ON668070; 540–740 bp; 1 bp difference), four ITS of rRNA genes (ON668071–ON668074; 800–920 bp; 3 bp differences), and four identical partial *cox1* gene (ON664917–ON664920; 400–440 bp) sequences were generated for *P. ilicis* n. sp. Some sequences of 18S (MW413740–MW413742), D2–D3 of 28S (MW413683–MW413684), and *cox1* (MW413706–MW413707) have also been published recently in Singh *et al*. (2021) as unidentified sequences of a *Paratylenchus* sp.

**Figure 4:**
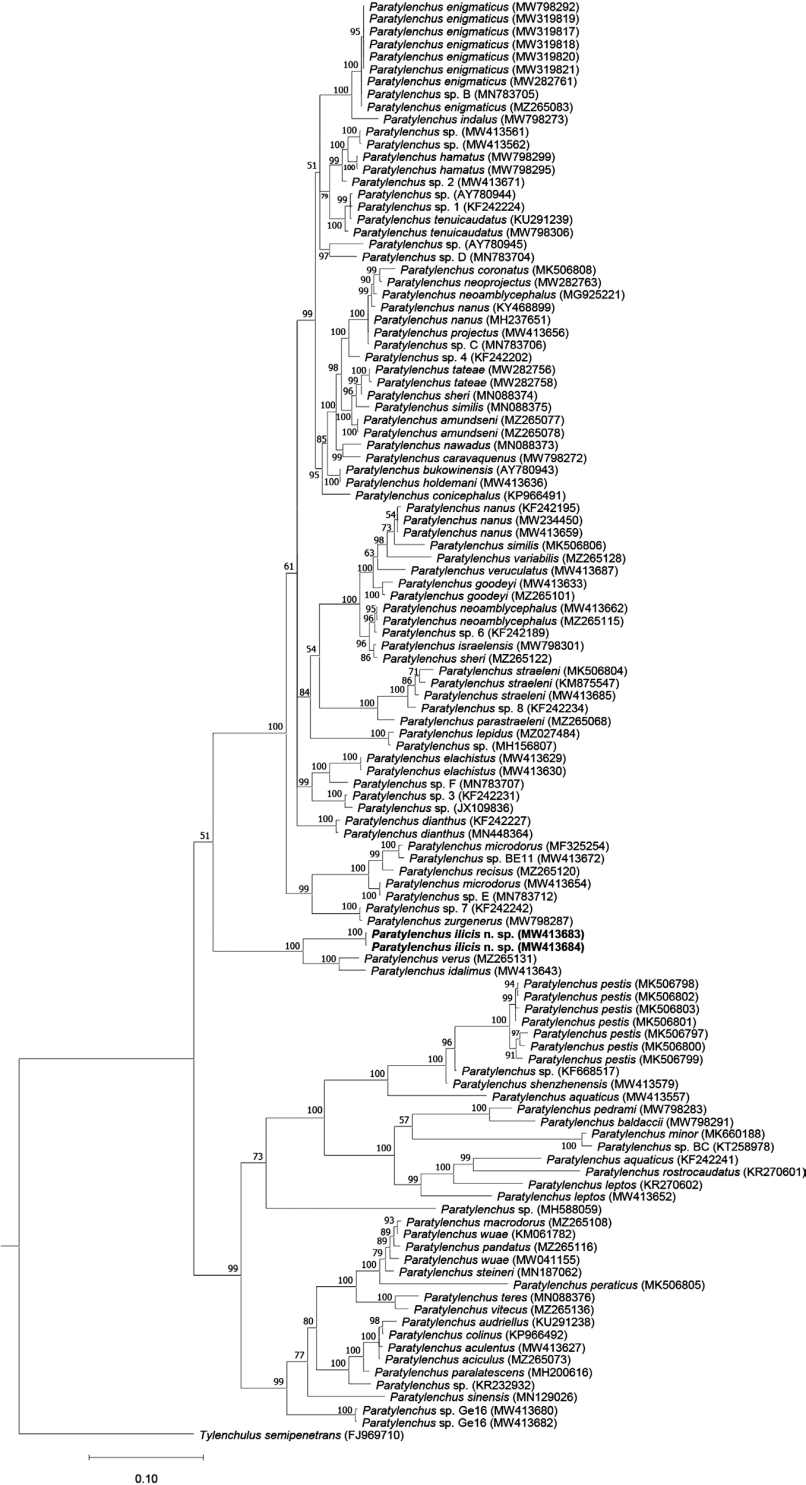
Phylogenetic tree generated using BI based on alignment of D2–D3 of 28S rRNA gene sequences of *Paratylenchus* species using the GTR + G + I nucleotide substitution model. Bayesian posterior probabilities (in percentage) are given next to each node and sequences of *Paratylenchus ilicis* n. sp. are highlighted. BI: Bayesian inference.

**Figure 5:**
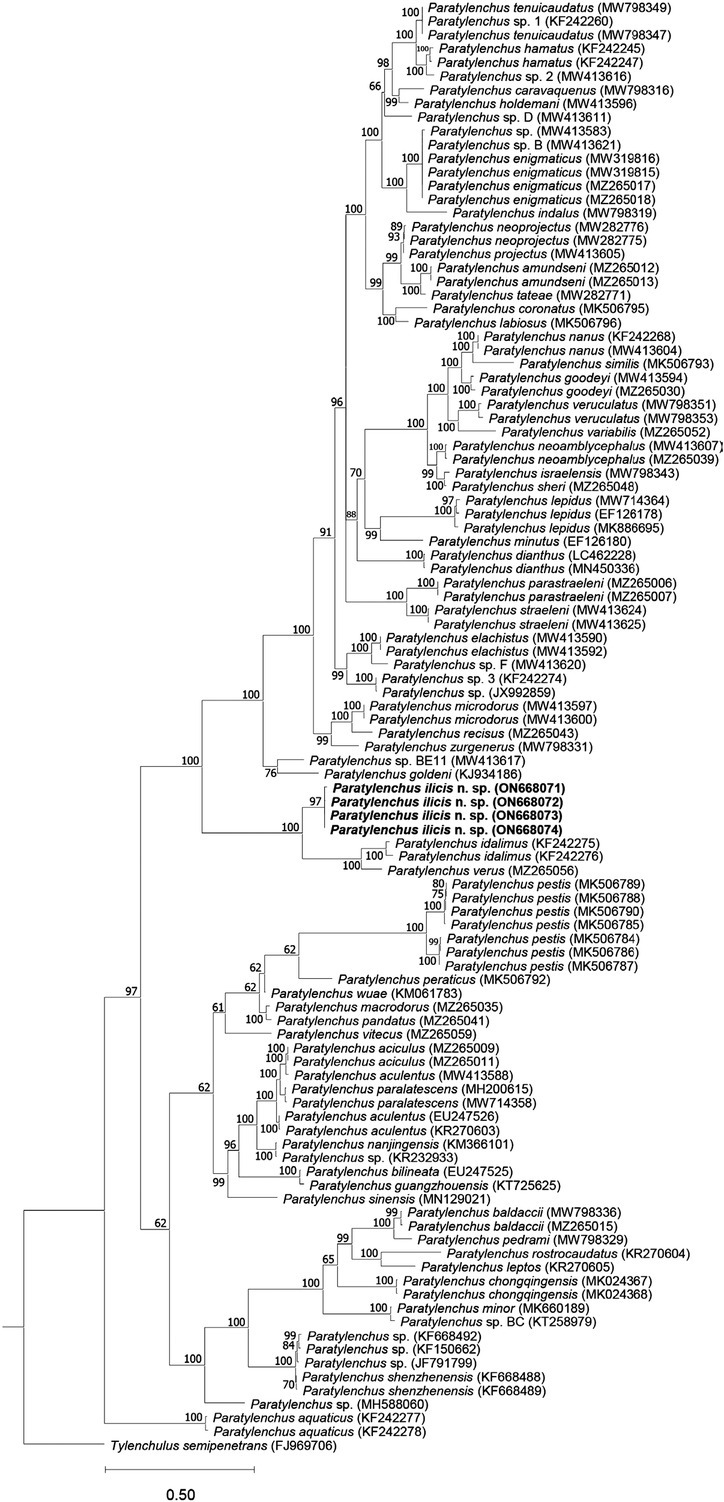
Phylogenetic tree generated using BI based on alignment of ITS rRNA gene sequences of *Paratylenchus* species using the GTR + G + I nucleotide substitution model. Bayesian posterior probabilities (in percentage) are given next to each node and sequences of *Paratylenchus ilicis* n. sp. are highlighted. BI: Bayesian inference.

**Figure 6:**
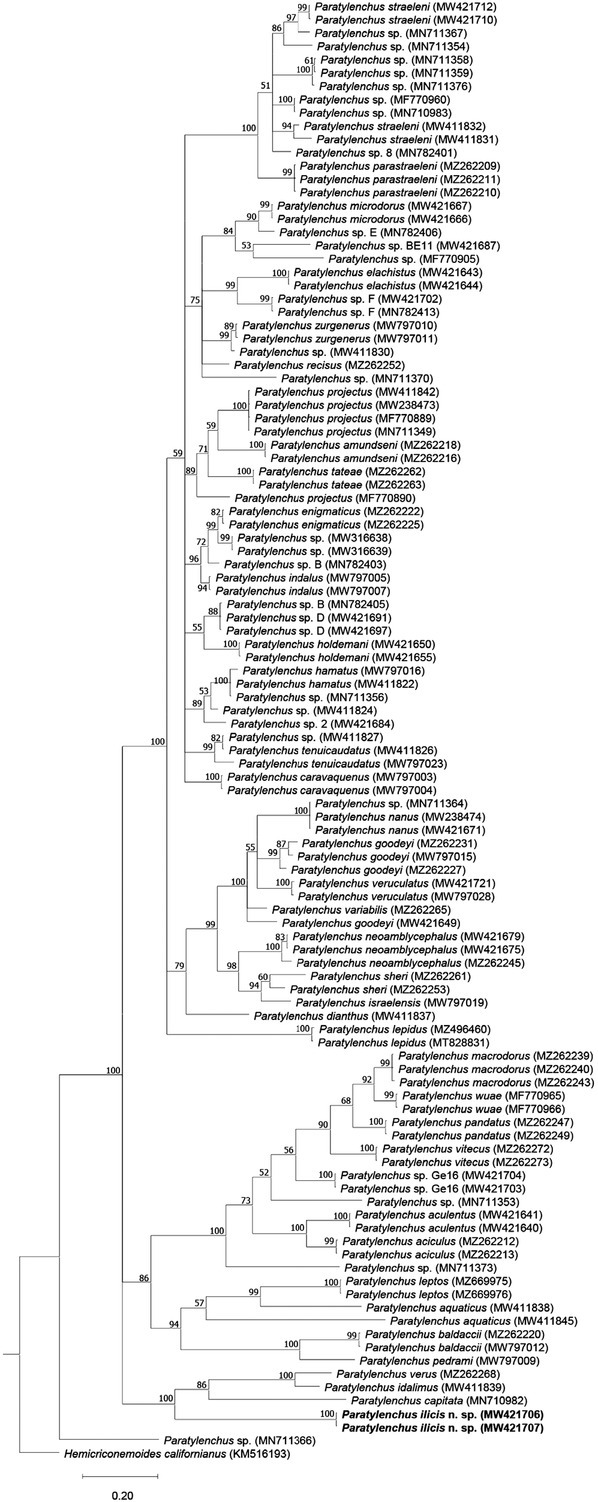
Phylogenetic tree generated using BI based on alignment of *cox1* gene sequences of *Paratylenchus* species using the GTR + G + I nucleotide substitution model. Bayesian posterior probabilities (in percentage) are given next to each node and sequences of *Paratylenchus ilicis* n. sp. are highlighted. BI: Bayesian inference.

The 18S sequences were found closest to *P*. *idalimus* sequences (MW413703–MW413704; 96.6% identity; 28 out of 820 bp differences), the D2–D3 sequences were found closest to *P*. *verus* sequences (MZ265132–MZ265133; 90.1% identity; 66 out of 669 bp differences), the ITS sequences were found closest to *P*. *nanus* Cobb, 1923 sequences (KY468904–KY468909; 86.5% identity; 48 out of 355 bp differences), and the *cox1* sequences were found closest to unknown *Paratylenchus* sp. sequences (MW421691–MW421699; 84.6% identity; 65 out of 422 bp differences). Based on both the D2–D3 and ITS trees (Figures 4 and 5), the new species has a maximally supported sister relation to a clade consisting of *P*. *idalimus* and *P*. *verus*. Based on the *cox1* tree (Figure 6), *P. ilicis* n. sp. forms a maximally supported clade with *P. idalimus* and *P*. *verus* (as in the other trees) and *P*. *capitata* (Adams and Eichenmuller, 1962) Siddiqi and Goodey, 1964.

## Evidences supporting synonymization of *Cacopaurus* with *Paratylenchus*

*Cacopaurus* was first synonymized with *Paratylenchus* by Goodey (1963) by shortly stating that there are no consistent distinguishing characters between the two except that adult females of the former are slightly swollen and become sessile in habit. The work by Mokaram Hesar *et al*. (2019) further delivered comprehensive morphological and molecular data of seven populations of *C*. *pestis* collected from different locations in northwest Iran in which morphometrics of obese females from all the populations and their molecular data of D2– D3 of 28S and ITS of rRNA genes were presented. Although no molecular data of the type population of *C*. *pestis* are available, the sequences from the Iranian populations should reliably represent *C*. *pestis* evidenced by both morphology and host of the Iranian populations, i.e., Persian walnut. The phylogenetic analyses of Mokaram Hesar *et al*. (2019) based on the two genes revealed the monophyly of all the seven Iranian populations; however, the species appeared embedded within a clade of *Paratylenchus*, suggesting that *Cacopaurus* may not be a valid genus. Nevertheless, the species status of *C*. *pestis* was still maintained in the paper.

In the current work, we presented morphological details of different life stages of *P. ilicis* n. sp. including juveniles, males, and slightly obese and fully obese females and their molecular data of rRNA and *cox1* gene fragments. This new species is morphologically very close to *C*. *pestis* in all life stages except for few differences (see above in the diagnosis and relationships section), but very clearly differentiated from the latter based on molecular data, habitat, and host. Importantly, the phylogenetic trees based on D2–D3 and ITS genes clearly show that “*Cacopaurus*” is polyphyletic, given that *P. ilicis* n. sp. is molecularly distantly related to *C*. *pestis*, also embedded in a *Paratylenchus* clade, and closely related to other *Paratylenchus* including *P*. *idalimus* and *P*. *verus*. Thus, these evidences support the view of Goodeyi (1963) that *Cacopaurus* is a junior synonym of *Paratylenchus*.

## Discussion

The pathogenicity of the majority of *Paratylenchus* species is unknown. However, several of them have been identified to cause substantial damage to various crops, especially when present in high density – for example, *P. dianthus* Jenkins and Taylor, 1956 was reported to retard growth of carnation in green houses (Jenkins and Taylor, 1956); *P. microdorus* Andrássy, 1959 delayed the growth of clover and lettuce (Andrássy, 1985); *P. hamatus* Thorne and Allen, 1950 negatively affected both quality and quantity of rose production (MacDonald, 1976); and *P. epacris* (Allen and Jensen, 1950) Goodey, 1963 was implicated in a disease of California black walnut in California (Allen and Jensen, 1950)*. Paratylenchus* are very small ectoparasitic nematodes with average body length between 0.25 mm and 0.30 mm, and often occur in high numbers in a population, but could also easily go undetected because of their small size (Siddiqi, 2000; Ghaderi *et al*., 2016). The discovery of *P. ilicis* n. sp. parasitizing on holly plant is important and concerning as the host is an important ornamental plant in temperate regions. This species was also detected in high numbers in our sample (>200 individuals in 100 ml of soil) with males and juveniles readily detected from soil sample by modified Baermann’s extraction method (Whitehead and Hemming, 1965); however, sessile and obese females could only be recovered from root materials by means of centrifugal flotation (van Bezooijen, 2006). The females could also be observed directly under a stereomicroscope as brownish, often dirt-like material, very close to the finer roots of the host. However, the life cycle and the pathogenicity of this new species, as well as the extent of its distribution and other host preferences, remain to be studied. So far, this species has not been detected elsewhere than at the type location (based on our recent surveys) and is therefore probably a rare nematode species in the Netherlands.

The synonymy of *Cacopaurus* and *Paratylenchus* proposed by Goodey (1963) is herein clearly supported by both morphological and molecular data. The morphology of juveniles, younger females, and males of both are highly similar (except that only the males of *P. pestis* and *P. ilicis* n. sp. have strongly reduced bursa). According to the generic key proposed by Siddiqi (2000), the main features separating *Cacopaurus* from *Paratylenchus* are that females of the former are obese-cylindroid and the post vulval region is shorter than vulval body width, while females of the later are vermiform (sometimes slightly obese, but never cylindroid) and the post vulval region is longer than vulval body width. However, in our study, the female vulval body width of the new species appeared to be influenced by the amount of obesity, and the post vulva region may or may not be shorter than the vulval body width. Therefore, these characteristics are not considered sufficient to treat the above as two separate genera. Additionally, our molecular data of multiple genes and resulting phylogenetic relationships clearly show that the two “*Cacopaurus*” species are imbedded in two different *Paratylenchus* clades and therefore, also from a phylogenetic point of view, *Cacopaurus* can be considered a junior synonym of *Paratylenchus*.
